# Ponto-Caspian mysid shrimp *Paramysis lacustris* has reached the Elbe estuary

**DOI:** 10.1038/s41598-025-04856-x

**Published:** 2025-06-04

**Authors:** Christine Schottmüller, Natascha Holl, Frank Suhling, Boris Schröder, C. Gabriel David

**Affiliations:** 1 Junior Research Group “Future Urban Coastlines”, Technische Universität Braunschweig, Braunschweig, 38106 Germany; 2 Landscape Ecology and Environmental Systems Analysis, Institute of Geoecology, Technische Universität Braunschweig, Langer Kamp 19c, 38106 Braunschweig, Germany; 3 Hydromechanics, Coastal and Ocean Engineering, Leichtweiß-Institute for Hydraulic Engineering and Water Resources, Technische Universität Braunschweig, Langer Kamp 19c, 38106 Braunschweig, Germany; 4 Plant Ecology, Institute of Ecology, Technische Universität Berlin, Rothenburgstraße 12, 12165 Berlin, Germany

**Keywords:** Paramysis, Mysida, Crustacea, Ponto-caspian neozoans, Estuary, First record, Invasive species, Population dynamics

## Abstract

As part of monitoring conducted alongside a river engineering project, the Ponto-Caspian mysid shrimp *Paramysis lacustris* (*P. lacustris*) was detected for the first time in the tidal Elbe in August 2023. Alongside *P. lacustris*, a second Ponto-Caspian mysid species, *Limnomysis benedeni* (*L. benedeni*), was also identified. The previous year’s investigation yielded no records of these mysids, suggesting that the species, last observed in 2019 in the Havel River in Saxony-Anhalt, are currently expanding into the tidal Elbe. In addition to addressing sampling aspects, we present the dispersal pathway of *P. lacustris* since its first recorded observation in 1882 and discuss the probable impact of the newly found species in the river Elbe based on former findings.

## Introduction

The native range of *Paramysis lacustris* (*P. lacustris*) spans the Ponto-Caspian region, encompassing the Black Sea, the Caspian Sea, the Sea of Azov, and the rivers and streams flowing into these basins^1^. Within this range, the species inhabits coastal areas, river estuaries, and lakes. Over the past five million years, major geoclimatic events have repeatedly altered the connections between Ponto-Caspian water bodies with varying salinities. These changes fostered the development of today’s unique brackish-water biodiversity, characterized by numerous adaptable and euryhaline species^2,3^.

Assuming that *P. lacustris* feeds on phytoplankton and detritus and can form large populations in mesotrophic lakes^4^, the species was deliberately introduced into Lithuanian waters in the 1960s. The goal was to enhance the local food chain by efficiently converting primary production into energy-rich biomass, thereby bolstering fish feed stocks^5^. Initially, it was released into the Kaunas Reservoir and the Curonian Lagoon in the Baltic Sea, followed by the Balkhash Lake in Kazakhstan^5,6^.

**Fig. 1:**
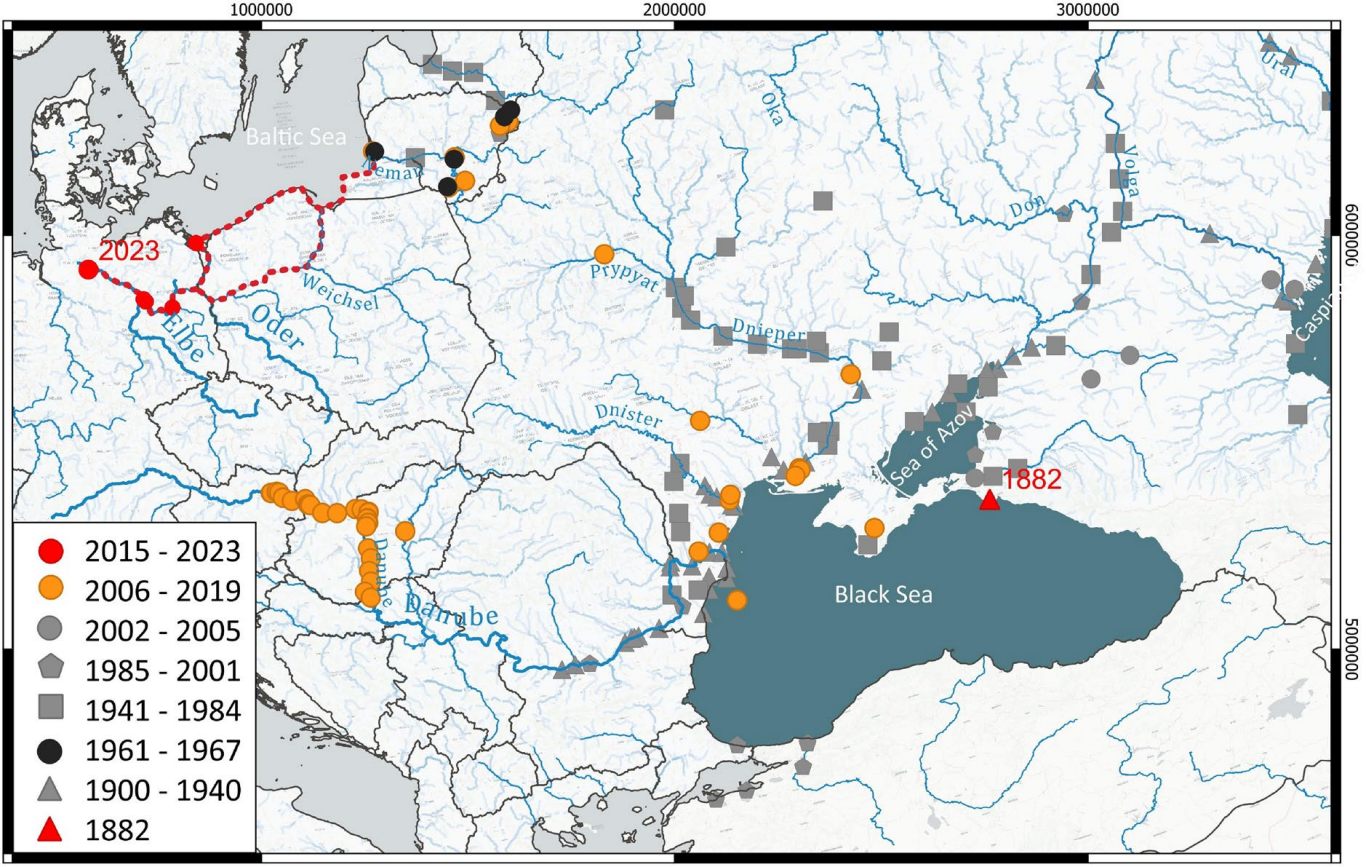
Dispersal paths of the mysid shrimp *Paramysis lacustris* (*P. lacustris*). Red triangle: Type locality^10^. More recent records overlay the older ones. The overview map is shown in the European Terrestrial Reference System 1989 (ETRS89) Universal Transverse Mercator (UTM) Zone 32N coordinate system (EPSG: 28532). The gray symbols display all records from the work of Wittmann^11^ and references therein. Black dots – Intentional introduction into Lithuanian waters in the 1960s^12^. Orange dots - records by Borza et al. 2019^13^. Dashed red line - Potential migration corridor into northern German waters via the Baltic Sea or the Vistula-Oder route. Red dots without year - Records in Germany by Zettler 2015^7^, Müller & Martens 2019^8^ and Hohmann & Zettler 2020^9^. Red dot with year - First record for the tidal Elbe in this study.The map was generated using QGIS version 3.32 using the river network shapefile by the United Nations (UN)’s Food and Agricultural Organization (FAO) global information system on water and agriculture AQUASTAT (Creative Commons BY-NC-SA 3.0 IGO; CC BY-NC-SA 3.0 IGO license) as well as *Natural Earth*’s lakes shapefile (available under Creative Commons Public Domain: CC PD List license).

From these locations, *P. lacustris* spread further westward, being recorded in Germany for the first time in 2013 in the Oder Lagoon near Kamminke^7^. It is believed that the species reached the Lower Havel near Berlin-Spandau via the Oder and the Oder-Havel Canal, constructed in 1914. It was documented there in 2017^8^ and two years later in a side channel of the Havel in northeastern Saxony-Anhalt^9^. Following the flow of the Havel, the species likely reached the Elbe at Gnevsdorf in Brandenburg. Fig. 1 illustrates potential dispersal pathways from the Ponto-Caspian region to the Elbe.

## Research site

**Fig. 2:**
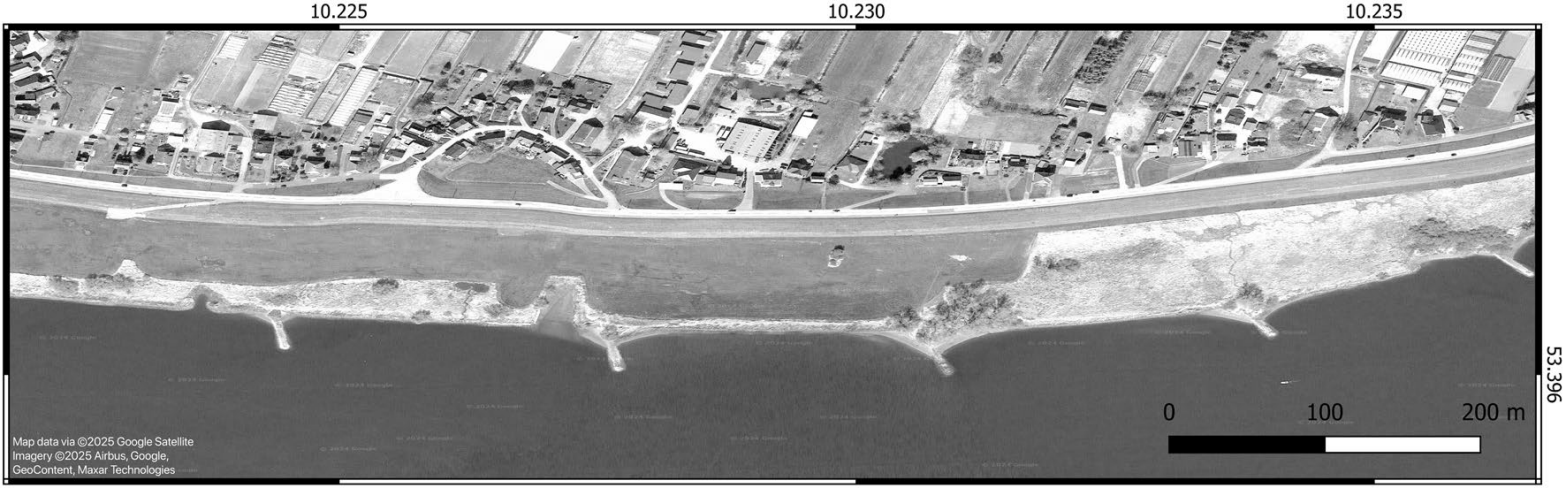
Study area in the tidal Elbe in the area of the Zollenspieker nature reserve, here at low tide. The overview map is shown in the World Geodetic System 84 (WGS84) coordinate system (EPSG: 4326). The map was generated using QGIS version 3.32 using a Google Satellite background map accessed (Maps Data ©2025) with data from Airbus Imagery of 6/9/2023 and Maxar Technologies, available for research without needing permission, according to the Google geo guidelines (as of March 2025).

The study area is located in the freshwater section of the tidal Elbe between river kilometers 595.5 and 596.7 (Fig. 2). The sampled 1,200-meter-long shoreline section lies adjacent to the district of Ost-Krauel and is part of the Zollenspieker Nature Reserve, protected under the Zollenspieker Nature Conservation Ordinance (*Verordnung über das Naturschutzgebiet Zollenspieker*, ZollenNatSchGebV HA) and the Flora-Fauna-Habitat Directive (*Fauna-Flora-Habitat-Richtlinie*, FFH).

At Zollenspieker, the shoreline features stone revetments and groynes extending into the river. A narrow strip of herbaceous riparian vegetation grows atop and behind the stone walls, transitioning into tall herbaceous fringes and floodplain forests. The groyne fields are classified as FFH habitat type “unvegetated river mudflats” (*Flusswatt ohne Vegetation*, FWO) and protected under §28(1) of the Hamburg Nature Conservation Act (*Hamburgisches Gesetz über Naturschutz und Landschaftspflege*, HmbNatSchG).

The groynes, spaced 200 meters apart, increase flow velocity in the river’s center while reducing it near the banks. This reduced flow allowed fine-grained and silty sediments, transported by floods and erosion in the navigational channel, to accumulate in the groyne fields. On one hand, this process has created flow-reduced areas near the groynes covered with anoxic mud, which often contains large amounts of undecomposed detritus. On the other hand, significant portions of the intertidal zone have silted up over time, resulting in the loss of biologically valuable shallow water zones.

To counteract this, three of the five groynes in the study area were notched to enhance hydrodynamics and promote sediment removal from the groyne fields. This measure was implemented in January 2023 by the Elbe Habitat Foundation^14^ and has been monitored scientifically since August 2022 by members of the early-career research group “Future Urban Coastlines” at TU Braunschweig.

During the sampling month, on August 16, 2023, the River Basin Community Elbe (RBC Elbe, *Flussgebietsgemeinschaft Elbe, FGG Elbe*) recorded the following chemical-physical parameters for water quality^15^: a Potential of Hydrogen (pH) value of 8.4, a water temperature of 19.4 °C, and an oxygen saturation of 112 % (8.3 mg$\text {L}^{-1}$). The oxygen saturation values exceeding 100 % and the elevated pH indicate increased activity of phototrophic organisms during the sampling period.

## Results

During the sampling of the groyne fields in the lower tidal Elbe on August 31, 2023, a total of 36 individuals of *P. lacustris* and a single specimen of *Limnomysis benedeni* (*L. benedeni*) were recorded (see Table 1).

**Table 1:** Site description of *Paramysis lacustris* (*P. lacustris*) in the upper tidal Elbe river with recorded Water Temperature (WT) and Salinity (PSU) During Sampling.

Date	Latitude	Longitude	Habitat	Count	WT (°C)	PSU
31.08.23	53.39692	10.23660	Water column near groyne	5	21.5	0.511
31.08.23	53.39633	10.23408	Water column near groyne	1	21.5	0.511
31.08.23	53.39628	10.22763	Tidal pool	31	21.5	0.511

**Fig. 3:**
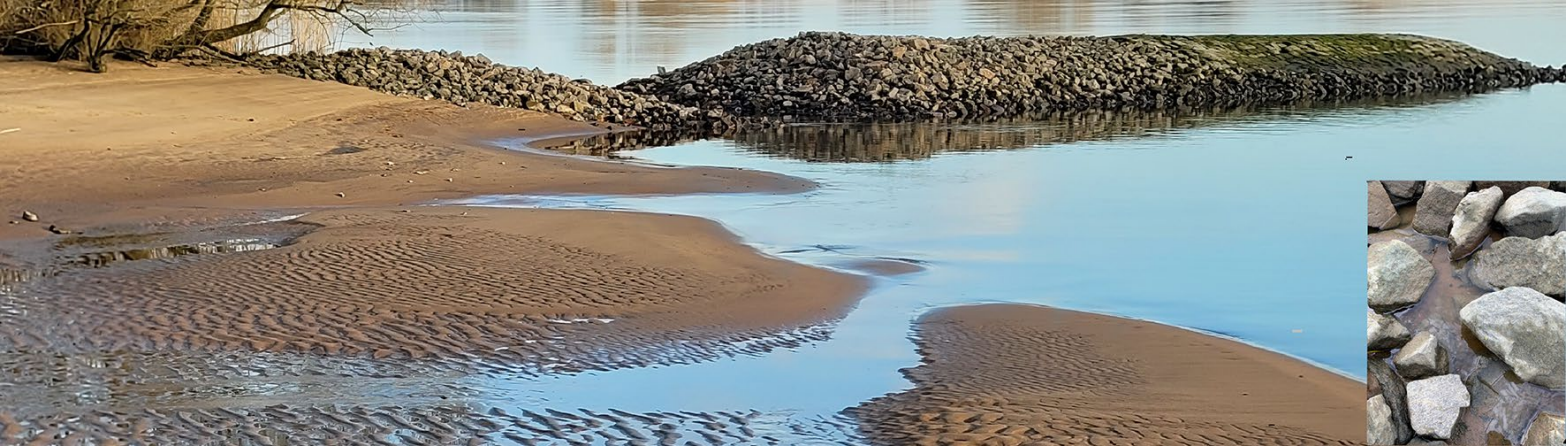
Notched groyne in the upper tidal Elbe near Ost-Krauel – Numerous mysid shrimps *Paramysis lacustris* (*P. lacustris*) were recorded in the water column near the groyne and in tidal ponds (bottom right in the picture). Photograph was provided to the authors courtesy of the Stiftung Lebensraum Elbe 2023.

The specimens were exclusively found in aquatic habitats near the groynes (Fig. 3). Thirty-one individuals were observed swimming between the reinforcement stones of a notched groyne, in small pools formed by the receding tide. No colonization of this habitat, characterized by hard substrate and coarse sand, by other species was detected.

**Fig. 4:**
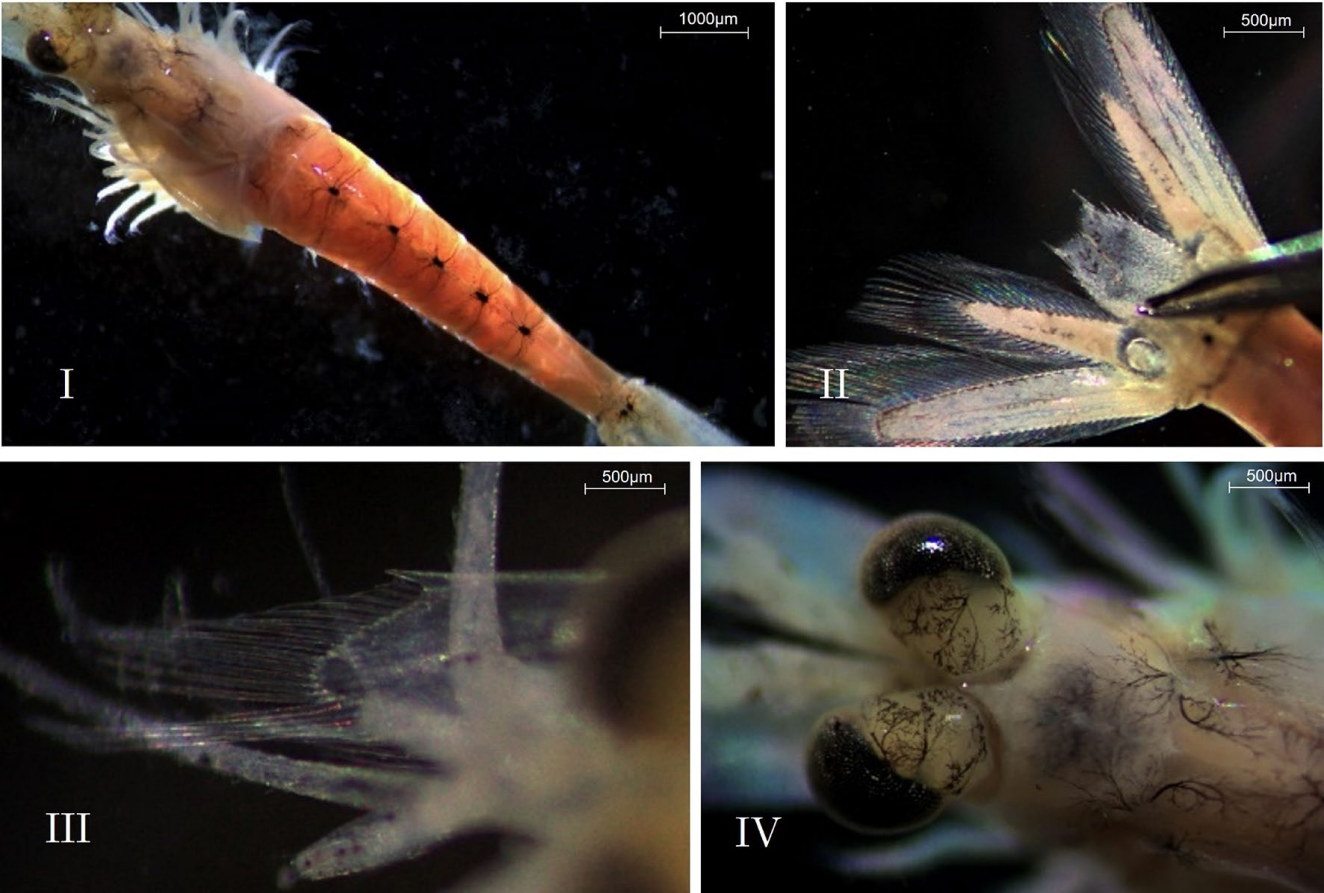
*Paramysis lacustris* (*P. lacustris*) and characteristic features. I: Ventral view of a female with characteristic dots on abdominal somites one to five, II: Telson with statocysts, III: Antennal scale with tooth, IV: Rostrum and eyes banded. Pictures were recorded with a Zeiss Stereo Microscope Stemi 305 trinocular EDU-Set (Carl Zeiss AG, Germany – see also methods).

In landing net samples taken at two different groynes, a total of five free-swimming *P. lacustris* (Fig. 4) and one *L. benedeni* were identified. Besides two chironomid larvae and three juvenile bivalves, no additional species were present in the net. Crustacean representatives were also found inhabiting sandy sediment as well as within and on deadwood (Table 2).

**Table 2:** List of Crustacea recorded in the study area with special focus on alien species and their origin. P-C : Ponto-Caspian, NW-A : Northwest Atlantic, NW-P: Northwest Pacific. The abundances of individuals in each habitat type are based on estimates.

Species	Alien species	Habitat Type	Ind/m^2^
Amphipoda			
*Chelicorophium robustum* (Sars, 1895)^16^	P-C	Deadwood/ Sand	16
*Dikerogammarus villosus* (Sowinsky, 1894)^17^	P-C	Deadwood	16
*Gammarus tigrinus* (Sexton, 1939)^18^	NW-A	Deadwood/ Sand	31
*Gammarus duebenii* (Liljeborg, 1852)^19^	–	Sand	2
*Gammarus zaddachi* (Sexton, 1912)^20^	–	Deadwood/ Sand	13
Decapoda			
*Eriocheir sinensis* (Milne Edwards, 1853)^21^	NW-P	Deadwood	11
Mysida			
*Limnomysis benedeni* (Czerniavsky, 1882)^10^	P-C	Stone revetment (submerged)	1
*Paramysis lacustris* (Czerniavsky, 1882)^10^	P-C	Stone revetment (submerged)	36

Among the eight crustacean species identified during the monitoring, 50% are of Ponto-Caspian origin. The presence of *Dreissena polymorpha* (*D. polymorpha*) in the study area with individuals inhabiting the sandy habitats near the low water line or colonizing on native duck mussel further highlights the influence of Ponto-Caspian species in the surveyed area.

## Discussion

For the Elbe, this study presents the first record of the mysid shrimp *P. lacustris*. The freshwater zone of the Elbe estuary featuring flow-reduced groyne fields indicates a suitable habitat for this species. *P. lacustris* prefers fine sediments such as sand, mud, and silt in standing or slow to moderately flowing water^1,22^.

*P. lacustris* is euryhaline, tolerating salinity fluctuations between 0 PSU to 13.8 PSU in its native range, which besides the Elbe includes the Black and Caspian Seas as well as major rivers such as the Danube, Volga, and Dnieper^11,23^.

Although *P. lacustris* can endure water temperatures as low as 0°C, seasonal storm surges and declining winter temperatures present challenges for its survival in the study area. In lakes and ponds, individuals retreat to deeper areas when temperatures drop below the species’ natural range^11^. However, in a dynamic river system like the Elbe, where deeper areas are near the navigation channel with flow velocities far exceeding the species’ habitat preferences, this strategy alone is unlikely to support successful establishment: Recent studies suggest that a combination of cold-tolerant traits – such as entering diapause or hibernation – enhances the ability of Ponto-Caspian invertebrates like *P. lacustris* to cope with very low temperature conditions^24^. This physiological adaptability to fluctuating temperature and salinity is a key factor for colonisation success of Ponto-Caspian species beyond their native range^1,23^.

Manmade waterways increase hydrologic connectivity thus enabling alien species to spread across European watersheds. This is epitomized by the Elbe River being a crucial component of Central Europe’s inland waterway network, linking the North Sea, Baltic Sea, Rhine, Danube, and Black Sea through an extensive system of rivers and canals. While this connectivity supports commerce, it also facilitates the dispersal of non-native aquatic invertebrates^25^. The saltwater-influenced zone of the Elbe estuary, in particular, now hosts an increasing number of alien macroinvertebrate species^23,26^. For instance, of the eight crustacean taxa identified in the study area, six were non-native with four of them originating from the Ponto-Caspian region. Of the two non-Ponto-Caspian aliens, the Chinese mitten crab *Eriocheir sinensis* (*E. sinensis*) was the only decapod found during our monitoring. Its range expansion is an example of human-mediated dispersal via maritime transport, which supposedly started in Germany in the port of Hamburg in the early decades of the last century and continued to rapidly distribute accross Europe^27^. As for *P. lacustris* in Lithuanian waters, the introduction of the North-American euryhaline *G. tigrinus* in German rivers was intentional: It was released into a river west of the Elbe (Werra) in the late 1950s to account for a declining population of the native gammarid populations due to salt pollution from mining activities^28^. From there, it expanded its range northeastward, possibly facilitated by the German Mittelland Canal, which connects major German rivers since 1938^29–31^. Although our sampling results showed the species vastly outnumbering the native amphipods *Gammarus zaddachi* (*G. zaddachi*) and *Gammarus duebenii* (*G. duebenii*), this is likely due to the habitat preferences of the latter, which left ecological niches open for colonization by alien species^26,30^. This factor needs to be taken into account when trying to assess possible ecological impacts of the arrival of *P. lacustris* in the Elbe.

Former observations indicate that *P. lacustris* can form large populations under favorable conditions^11,12^. The introduction of the Ponto-Caspian mysid shrimp *P. lacustris* into European mesotrophic lakes and reservoirs has led to significant alterations in local food webs^5^. Originally assumed to feed primarily on phytoplankton and detritus, *P. lacustris* has demonstrated omnivorous feeding habits, preying on various invertebrates, including mesozooplankton species. Its dietary flexibility enables *P. lacustris* to occupy a higher trophic level, thereby extending food chain length and potentially affecting the trophic position of native planktivorous fish, such as the European perch^4,5^, *Perca fluviatilis* (*P. fluviatilis*). Thus the establishment of *P. lacustris* may lead to increased competition for food resources among native zooplanktivorous species and could entail negative effects on the overall energy flow within the ecosystem. Yet, the Elbe river is a heavily modified waterway that has undergone extensive alterations to its riverbanks and habitats, and conclusions from research in lakes and water reservoirs can probably not be drawn directly. Comparing our current observations to biological river water quality assessments based on the European Water Framework Directive^32^, we found that species composition and abundance is classified as only moderate in our study area, with only four Crustacean species found in 2018^33^. Thus, in the context of the Elbe Habitat Foundation’s objective to create suitable habitats for fish and lampreys within groyne fields, the occurrence of *P. lacustris* as an additional energy-rich food source may be considered beneficial.

## Methods

The sampling was conducted on behalf of the Elbe Habitat Foundation (*Stiftung Lebensraum Elbe*) to evaluate the impact of the construction measures, particularly regarding the development of suitable habitats for fish and lampreys within the groyne fields. In late summer 2023, a total of 17 samples were collected from three different habitat types: sand, deadwood, and stone revetments, to assess the communities inhabiting the groyne fields.

The sandy, partially silty soft substrate along the low-water line was sampled at ten locations, each covering an area of 25 cm x 25 cm, using a 500 $\upmu$m mesh sieve. At three additional locations, deadwood components were collected from an equivalent area using tweezers. The remaining four samples were taken from aquatic habitats between and adjacent to the immobile stones of the groynes. In these cases, stones were brushed, and a simple hand net with a mesh size of 500 $\upmu$m and a frame length of 25 cm was used to perform four net sweeps.

For all habitat types, a preliminary live sorting was conducted in the field, with only a small number of individuals preserved for further identification in the laboratory.

We used a Zeiss Stereo Microscope Stemi 305 trinocular EDU-Set (Carl Zeiss AG, Germany) equipped with an eyepiece 16$\times$/14 Br. foc and an Objective 2.0$\times$ FWD 43 mm front optics system 3 for Stemi 305. Images were captured using an Axiocam 208 color microscope camera (USB3, 8 MP, 1/2.1 in). Imaging and analysis were conducted using the free version of Zen Core 3.6 software (Carl Zeiss AG).

## Data Availability

The point shapefile in Figure 1 was created in QGIS compiling the information and coordinates in the publications cited in the figure captions. The points and can be made available upon reasonable request by Christine Schottmüller, ORCID 0009-0009-1465-237X. All other data generated or analyzed during this study are included in the published article.
